# In-Use Stability and Device Compatibility Define Clinically Actionable Handling Limits for a GMP-Produced Attenuated *Listeria monocytogenes* Vaccine Expressing GUCY2C

**DOI:** 10.3390/vaccines14050461

**Published:** 2026-05-21

**Authors:** Jagmohan Singh, Taranjot Johar, Vannessa Scully, Scott A. Waldman, Babar Bashir, Adam E. Snook

**Affiliations:** 1 Department of Pharmacology, Physiology, and Cancer Biology, Thomas Jefferson University, Philadelphia, PA 19107, USA; jagmohan.singh@jefferson.edu (J.S.); scott.waldman@jefferson.edu (S.A.W.); 2 Investigational Drug Service, Thomas Jefferson University Hospital, Philadelphia, PA 19107, USA; vannessa.scully@jefferson.edu; 3 Department of Medical Oncology, Thomas Jefferson University, Philadelphia, PA 19107, USA; babar.bashir@jefferson.edu

**Keywords:** guanylyl cyclase C (GUCY2C), *Listeria monocytogenes*, cancer vaccine, in-use stability

## Abstract

**Background:** Live-attenuated *Listeria monocytogenes* (Lm) vectors are a clinically validated cancer immunotherapy platform, but translation requires reproducible, clinically realistic workflows for dose preparation and infusion. For live bacterial products, in-use stability and device compatibility can drive dose variability through adsorption, settling, and device losses. **Methods:** We developed and GMP-manufactured an attenuated Lm vaccine expressing human GUCY2C (Lm-GUCY2C) and performed translational characterization, including construct verification and immunogenicity readouts, and defined the administration-focused in-use stability and device compatibility. Post-thaw stability was assessed in primary cryovials and during preparation and delivery from 250 mL saline infusion bags using standard clinical devices (syringes/needles, filter-free IV tubing) and OnGuard2 closed-system components. Samples were collected over 24 h at room temperature, and viable Lm-GUCY2C were quantified by CFU recovery. **Results:** Lm-GUCY2C remained stable in thawed cryovials for 24 h with no significant CFU loss. High-dose infusion bags (3 × 10^9^ CFU/bag) maintained CFU recovery through 6 h, whereas low-dose bags (3 × 10^8^ CFU/bag) exhibited significant losses beginning at 3 h, supporting a practical in-use window of up to 2 h for low-dose preparations. OnGuard2 intravenous (i.v.) connectors did not measurably affect CFU recovery, while OnGuard2 vial adapters reduced recovery. **Conclusions:** This work provides an end-to-end, translationally focused characterization of a GMP-manufactured Lm cancer vaccine, including clinically actionable in-use handling constraints and device compatibility. These data define preparation and administration guardrails (notably, time-to-infusion limits for low-dose bag preparations) that can improve dose accuracy and reproducibility in clinical testing.

## 1. Introduction

Guanylyl cyclase C (GUCY2C) is a membrane receptor normally confined to intestinal epithelial cells but broadly overexpressed across primary and metastatic colorectal cancer (CRC) lesions, making it an attractive and emerging target for CRC immunotherapy using antibody–drug conjugates [1,2,3,4,5,6], bispecific T-cell engagers [7,8], CAR-T cells [9,10,11], and cancer vaccines [12,13].

Indeed, we identified GUCY2C as an immunotherapeutic target [14] and demonstrated that a replication-incompetent serotype 5 adenoviral vector expressing GUCY2C (Ad5-GUCY2C-PADRE) safely primes GUCY2C-specific T-cell responses in CRC patients [12]. However, Ad5 vectors, including Ad5-GUCY2C-PADRE, are sensitive to pre-existing neutralizing antibodies (NAbs) induced by natural exposure to adenovirus [15].

Further, repeated adenoviral vector administrations induce de novo NAb responses, limiting the effectiveness of repeated adenoviral vector administrations and the durability of vaccine-induced immunity [16]. Live bacterial vectors, such as *Listeria monocytogenes* (Lm), have emerged as alternative candidates reflecting their intrinsic immunostimulatory properties and ability to deliver antigens directly to antigen-presenting cells (APCs) [17,18,19]. Lm is a Gram-positive, facultative intracellular bacterium that naturally infects macrophages and dendritic cells through phagocytosis. Following phagosomal escape via the Lm protein listeriolysin O (LLO), Lm replicates in the cytosol, enabling efficient antigen presentation via both MHC class I and class II pathways and induction of CD8^+^ cytotoxic T lymphocyte (CTL) responses.

Attenuation by deletion of virulence genes such as *actA*, required for intracellular actin-based motility and cell-to-cell spread, and *inlB*, required for invasion of non-phagocytic cells, particularly hepatocytes, via the Met receptor, reduces pathogenicity while preserving immunogenicity, yielding vaccine strains suitable for clinical use [20]. Further, Lm vectors are not sensitive to pre-existing immunity, allowing for repeated administrations to provide boosts and long-lasting immunity [21]. However, when Lm is used as a standalone vaccine to prime immune responses against a self-antigen such as GUCY2C, the strong T-cell responses directed against immunodominant Lm proteins (including LLO, p60, and Mpl) competitively suppress the development of T-cell responses to the weaker, vaccine-encoded tumor antigen [22]. This immunodominance hierarchy limits the ability of Lm vectors to independently prime anti-tumor immunity.

To overcome these limitations, we developed a heterologous prime-boost immunization strategy targeting GUCY2C in which a chimeric adenoviral vector possessing the serotype 35 fiber (Ad5.F35), which evades pre-existing NAbs by utilizing a different cellular entry pathway, first primes robust T-cell responses [15]. Subsequent boosting with an attenuated Lm then selectively amplifies the already-established GUCY2C-specific memory T-cell population, as memory T cells expand preferentially over naive T cells in the competitive immunodominance environment of Lm infection [21]. In preclinical studies, Lm-GUCY2C repeatedly and effectively amplified GUCY2C-specific CD8^+^ T cell responses primed by Ad5.F35-GUCY2C-PADRE, circumventing the NAb-mediated attrition that limits repeated adenoviral dosing and immunodominance of Lm antigens [21]. A dose-finding study of Ad5.F35-GUCY2C-PADRE confirmed its ability to overcome pre-existing adenoviral immunity to induce robust immune responses in CRC without safety concerns [13]. Based on these findings, Lm-GUCY2C is advancing to Phase I clinical testing as a boost to the Ad5.F35-GUCY2C-PADRE prime in patients with metastatic CRC (NCT07417488).

A critical but often underappreciated prerequisite for clinical use of live bacterial vaccines is systematic characterization of their in-use handling properties. In contrast to recombinant protein subunit, nucleic acid, or viral vector vaccines, live bacterial products are highly susceptible to losses due to adsorption to plastic surfaces, physical filtration, and time-dependent changes in viability under ambient clinical holding conditions [23,24,25,26].

The specific formulation matrix, device materials, and administration timeline can all affect the delivered dose. Despite the clinical pipeline of Lm-based cancer vaccines, no comprehensive data exist describing post-thaw in-use stability or compatibility with standard intravenous (i.v.) administration devices for any Lm vaccine product—a gap that creates uncertainty for clinical protocol development and regulatory submissions. Here, we describe the GMP manufacturing of Lm-GUCY2C and its translational characterization, including construct integrity verification, in vitro expression confirmation, in vivo immunogenicity in mice, and systematic in-use stability and i.v. device compatibility characterization, the first for a live GMP-grade Lm cancer vaccine. Our data establish clinically actionable time limits for dose preparation and administration and identify compatible and incompatible device components, directly informing clinical testing of Lm-GUCY2C and similar Lm products.

## 2. Materials and Methods

### 2.1. Plasmid Construction and Synthesis of ActA-hGUCY2C

The extracellular domain of human GUCY2C (hGUCY2C_23–429_) was codon-optimized for expression in Lm using the Java Codon Adaptation Tool [27] and synthesized as a fusion construct placed under control of the *actA* promoter, downstream of a modified first 100 amino acids of ActA (ActAN100*) [28] and a synthetic enhancer sequence (Syn18 × 5) [29]. The resulting ActA-hGUCY2C cassette was synthesized by Genscript (Piscataway, NJ, USA) in the pUC57-Kan-mini vector (Project ID U0296HB160-2) and confirmed by restriction digestion and Sanger sequencing. The construct was propagated in chemically competent *E. coli* (Thermo Fisher Scientific, Waltham, MA, USA; Cat. No. C404003), and glycerol stocks were maintained at −80 °C.

### 2.2. Subcloning into the pPL2 Chromosomal Integration Vector

Plasmid DNA was isolated from a 10 mL LB culture and digested with KpnI and NotI. The liberated ActA-hGUCY2C expression cassette fragment was gel-purified and ligated into KpnI/NotI-cut, alkaline phosphatase-treated pPL2 [30], a chromosomal integration vector targeting the tRNA^Arg^ locus of Lm. Ligation products were transformed into TOP10 *E. coli* and plated on LB-agar containing chloramphenicol (7.5 μg/mL). Colonies were screened by PCR, and three PCR-positive clones were confirmed by Sanger sequencing to contain the intact ActA-hGUCY2C insert, free of frameshifts or truncations.

### 2.3. Chromosomal Integration into ΔactAΔinlB Lm by Conjugation

The attenuated ΔactAΔinlB Lm strain (ATCC PTA-5562) was used as the parental strain for all Lm-GUCY2C constructs. Sequence-verified pPL2-ActA-hGUCY2C plasmid (1 μg) was electroporated into SM10 λpir *E. coli* with a single 17 ms pulse at 500 V (ECM 830 Electroporator; Harvard Apparatus, Holliston, MA, USA). Transformants were selected on chloramphenicol LB-agar. Conjugation was performed by co-culturing overnight liquid cultures of plasmid-bearing SM10 and ΔactAΔinlB Lm on antibiotic-free BHI agar, then selecting transconjugants on BHI-agar containing chloramphenicol (7.5 μg/mL) and streptomycin (200 μg/mL). Three independent colonies were PCR-screened with five primer pairs specific to the integrated ActA-hGUCY2C cassette and one pair serving as an Lm-specific positive control. Primer sequences are provided in Supplementary Table S1.

### 2.4. Identity Testing and Whole-Genome Sequencing

PCR assays distinguishing wild-type Lm from ΔactAΔinlB Lm were established using primers targeting the *actA* and *inlB* loci (Supplementary Table S1). Genomic DNA was isolated from overnight BHI cultures by phenol/chloroform extraction and diluted to 25 ng/μL. The lead Lm-GUCY2C clone (clone #1) underwent whole-genome sequencing (WGS) at CD Genomics (Shirley, NY, USA). Cell pellets from duplicate 10 mL cultures were washed twice with PBS, flash-frozen in liquid nitrogen, and shipped on dry ice. WGS reads were aligned to the expected construct and the parental Lm genome to confirm integration-site fidelity, deletion of *actA* and *inlB*, and to identify off-target single-nucleotide variants (SNVs).

### 2.5. Cell Culture, Intracellular Infection, and Western Blot

Murine macrophage-like J774A.1 cells (ATCC TIB-67) were maintained in DMEM supplemented with 10% FBS and penicillin/streptomycin (100 U/mL; 100 μg/mL) at 37 °C, 5% CO_2_. For infection, 5 × 10^5^ cells/well were seeded in 6-well plates and cultured overnight. Lm-GUCY2C or empty-vector control Lm were thawed, washed twice in PBS, and resuspended to 1 × 10^9^ CFU/mL. Cells were infected at a multiplicity of infection (MOI) of 10:1 for 1 h at 37 °C, followed by two PBS washes and addition of gentamicin-supplemented medium (10 μg/mL) for 5 h to eliminate extracellular bacteria. Cell lysates were harvested in RIPA buffer with protease inhibitors and analyzed by 4–12% SDS-PAGE. Western blots were probed with anti-hGUCY2C antibody MS20 (2 μg/mL) and anti-LLO antibody (1:500; Abcam, Waltham, MA, USA; Cat. no. ab200538), followed by HRP-conjugated secondary antibody (1:25,000; Jackson ImmunoResearch, West Grove, PA, USA; Cat. No. 115-035-062).

### 2.6. Animal Studies and IFNγ ELISpot Assay

All animal procedures were approved by the Thomas Jefferson University Institutional Animal Care and Use Committee (IACUC; Protocol 01956, Approved 4 February 2022). Six- to eight-week-old BALB/cJ mice (Jackson Laboratory, Bar Harbor, ME, USA) were randomized into groups of 10 (5 male, 5 female based on prior studies [22]) and received a single intraperitoneal (i.p.) injection of 1 × 10^7^ CFU of Lm-GUCY2C or parental control Lm in 200 μL sterile PBS. Cage location was not randomized or rotated during the study, and the order of treatments and measurements followed an unblinded, fixed group-by-group sequence. Given the small number of cohorts (2), their small sizes (n = 9), and the absence of subjective assessments, these potential confounders were not explicitly controlled. On day 7, spleens were harvested aseptically and processed into single-cell suspensions by mechanical disruption followed by red blood cell lysis.

GUCY2C-specific T cell responses were quantified by IFNγ ELISpot (Cellular Technology Limited, Cleveland, OH, USA) according to the manufacturer’s protocol for each animal. Splenocytes were plated in CTL-TEST medium with 0.1% DMSO and stimulated overnight with 2 μg/mL of a 15-mer overlapping hGUCY2C_1–429_ peptide library [12] (JPT Peptide Technologies, Berlin, Germany), 10 μg/mL LLO_91–99_ peptide (positive control), or 0.1% DMSO (negative control). Spot-forming cells (SFCs) were quantified using an ImmunoSpot S6 Universal Analyzer (Cellular Technology Limited). GUCY2C- and LLO- specific responses were calculated by subtracting mean DMSO SFC counts from peptide-stimulated SFC counts. No animals were excluded from the analyses.

### 2.7. GMP Manufacturing

Following confirmation of construct integrity and preclinical immunogenicity, the lead Lm-GUCY2C clone was transferred to Waisman Biomanufacturing (Madison, WI, USA) for production under current Good Manufacturing Practice (cGMP) conditions. Prior to full GMP production, pilot-scale process development was conducted across two engineering runs to establish fermentation kinetics, tangential flow filtration (TFF) parameters, and fill conditions. Seed cultures were prepared by inoculating 100 µL of research cell bank (RCB) material into 100 mL Seed Medium and incubating at 37 ± 2 °C, 225 RPM. Pilot runs evaluated 12 and 16 h seed incubation windows; both produced adequate growth, and a 12 h seed incubation was selected for GMP production of a single lot of Lm-GUCY2C. Fed-batch fermentation was conducted at 37 ± 1 °C, 950 RPM, 1 Lpm air/O_2_ sparging, pH 7.2 ± 0.1, with dissolved oxygen controlled via a pO_2_ cascade set to 50%. Harvest was initiated when OD_600_ reached ≥ 2.0 or the specific growth rate (µ) fell below 0.25 h^−1^, criteria established during pilot runs as typically being met within 2.5–3.5 h of effective fermentation time. The culture was cooled to 2–8 °C and processed by TFF at feed pump rates of 300–600 mL/min, maintaining inlet pressure below 10 psig and permeate flux of 20–75 L/m^2^/h, with diafiltration against 5–7 diavolumes of PBS/10% glycerol prior to concentration to final harvest volume. The bulk retentate was formulated in PBS supplemented with 10% glycerol at a nominal concentration of 1.2 × 10^9^ CFU/mL and filled at 1.2 mL per vial in 2 mL glass cryovials, sealed by aluminum crimp, and stored at −80 °C. Release testing confirmed identity, purity, potency (CFU), and sterility in accordance with regulatory requirements for Phase I/II clinical use.

### 2.8. In-Use Stability Testing in Cryovials

Stability testing was conducted at room temperature (~22 °C) under aseptic conditions in a BSL-2 biosafety cabinet to simulate expected clinical dose preparation conditions. Details for all materials used for stability and device compatibility testing are included in Supplementary Table S2. Cryovials were thawed at room temperature for 8 min. Samples (100 μL) were withdrawn aseptically at 0, 0.5, 1, 2, 3, 6, and 24 h post-thaw—individual vials were repeat tested across the time course, rather than different vials at each time point. Duplicate ten-fold serial dilutions were prepared in PBS/10% glycerol (the formulation buffer), and 100 μL of the 10^−6^ and 10^−7^ dilutions were plated onto tryptic soy agar (TSA) plates in triplicate. Plates were incubated at 37 °C for 48 h, and colonies were enumerated using Onepetri version 1.1.3 (McGill University, Montreal, QC, Canada) and Promega Colony Counter version 1.5 (Promega, Madison, WI, USA) software to determine titers. All experiments were performed in biological triplicate (ex, 3 cryovials or 3 prepared saline bags).

### 2.9. In-Use Stability Testing in Saline Infusion Bags

Drug product was transferred aseptically from thawed cryovials into 250 mL 0.9% sodium chloride injection bags (Baxter Healthcare, Deerfield, IL, USA) via sterile syringe and needle, at either high dose (3 × 10^9^ CFU/bag; using a 3 mL Luer-lock syringe) or low dose (3 × 10^8^ CFU/bag; using a 1 mL Luer-lock syringe), corresponding to the anticipated clinical dose range. The drug product was pooled by collecting it into a single syringe before addition to the bag. Bags were gently hand-mixed for 2 min to ensure homogeneous distribution. A filter-free i.v. infusion line was attached and primed. Prior to each sampling time point, 20 mL of vaccine-containing saline was discarded to flush any settled bacteria from the tubing, and 1 mL aliquots were then collected. Samples were supplemented with 200 μL PBS/10% glycerol (formulation buffer), and 10-fold serial dilutions were plated as described above. Time points included 0, 0.5, 1, 2, 3, 6, and 24 h after bag preparation and were sampled from the same bags at each time point. Titers (CFU/mL) were determined and used to calculate the delivered dose (CFU/mL × bag volume).

### 2.10. Closed-System Transfer Device Compatibility Testing

Compatibility with OnGuard^®^2 closed-system transfer devices (B. Braun, Bethlehem, PA, USA) was evaluated using two components: (1) the Syringe Adaptor Lock (SAL), used to inject vaccine into the infusion bag, and (2) the Luer Lock Adaptor (LLA), used to connect the i.v. line. Low-dose bags (3 × 10^8^ CFU) were prepared and sampled as above. Additionally, the OnGuard^®^2 Vial Adaptor, which incorporates an activated charcoal filter and a 0.22 μm membrane filter, was evaluated by routing vaccine withdrawal through the adaptor and assessing CFU recovery relative to standard needle-and-syringe transfer.

### 2.11. Data Analysis and Manuscript Preparation

Data were plotted and analyzed using GraphPad Prism version 10. CFU values at each time point were compared to the 0 h baseline using one-way ANOVA with Bonferroni correction for multiple comparisons. Statistical significance thresholds: * *p* < 0.05; ** *p* < 0.01; *** *p* < 0.001. All experiments were performed in biological triplicate, and data are presented as the mean of replicates ± SEM.

## 3. Results

### 3.1. Construct Design and Genetic Verification of Lm-GUCY2C

**Figure 1:**
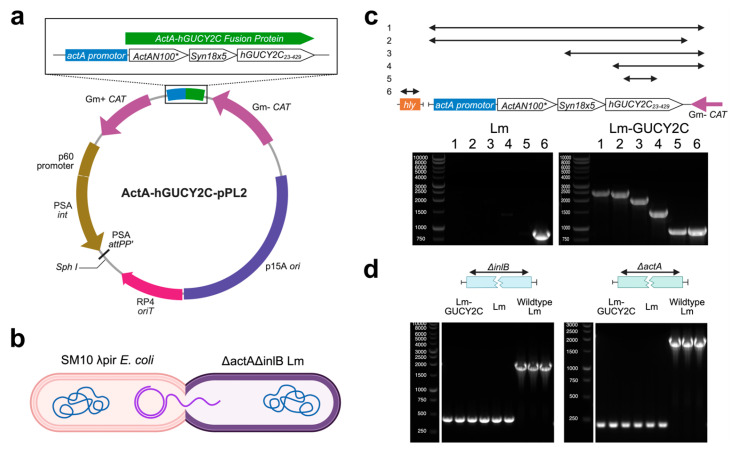
Lm- GUCY2C Generation. (**a**,**b**) Schematics depicting the ActA-hGUCY2C expression cassette cloned into the pPL2 plasmid and delivery of ActA-hGUCY2C-pPL2 to ΔactA/ΔinlB Lm by transconjugation from SM10 λpir *E. coli.* (**c**) PCR results using 5 different primer pairs specific to the ActA-hGUCY2C expression cassette (#1–#5) and a pair specific to an Lm gene serving as an internal positive control (#6; *hly*). The representative Lm-hGUCY2C clone (**right**) produced appropriate bands for all PCRs, while the negative control parental ΔactA/ΔinlB Lm (**left**) produced only the expected band in PCR #6. (**d**) inlB (**left**) and actA (**right**) PCRs indicate truncated products in Lm-GUCY2C and in parental ΔactA/ΔinlB Lm, while wildtype Lm possessed the larger wild-type sequences. Schematics in (**c**,**d**) indicate the PCR amplicons. Primer sequences are in Supplementary Table S1.

To generate an Lm-based cancer vaccine targeting GUCY2C for clinical use, the extracellular domain of human GUCY2C (hGUCY2C_23–429_) was placed under transcriptional control of the *actA* promoter as a fusion with ActAN100* and a synthetic enhancer sequence, coupling antigen expression to the Lm intracellular life cycle (Figure 1a). This cassette was subcloned into the pPL2 chromosomal integration vector (Figure 1a) and introduced into the attenuated ΔactAΔinlB Lm parental strain by conjugation (Figure 1b).

PCR screening of transconjugant colonies using five independent primer pairs confirmed integration of the ActA-hGUCY2C cassette in all positive clones (Figure 1c and Figure S1a). Sanger sequencing of gel-extracted PCR products from the lead clone (clone #1, selected from three sequence-verified clones based on PCR screening and Sanger sequencing confirmation) showed 100% identity to the expected ActA-hGUCY2C insert sequence, with ≥2× coverage across the full cassette. Identity testing of the *actA* and *inlB* loci confirmed that both deletions were retained in Lm-GUCY2C, producing truncated PCR products at the expected sizes compared to the larger wild-type bands, consistent with the attenuated parental strain (Figure 1d and Figure S1b). To provide a comprehensive genetic characterization required for clinical use, the lead clone underwent WGS (Supplementary Figure S2). Read alignment confirmed precise insertion of the hGUCY2C expression cassette into the intended pPL2 integration locus, flanked by expected chromosomal sequences, with no evidence of rearrangements or unintended duplications. The ΔactA and ΔinlB deletions were confirmed across the genome. SNV analysis identified a small number of single-nucleotide changes outside the targeted integration region; however, none were located within the hGUCY2C expression cassette, the *actA* deletion site, or the *inlB* deletion site. Together, these data confirm that the cGMP Lm-GUCY2C product is genetically stable, carries the intended safety deletions, and accurately encodes the designed antigen cassette.

### 3.2. Lm-GUCY2C Expresses and Secretes the ActA-GUCY2C Fusion Protein in Macrophages

**Figure 2:**
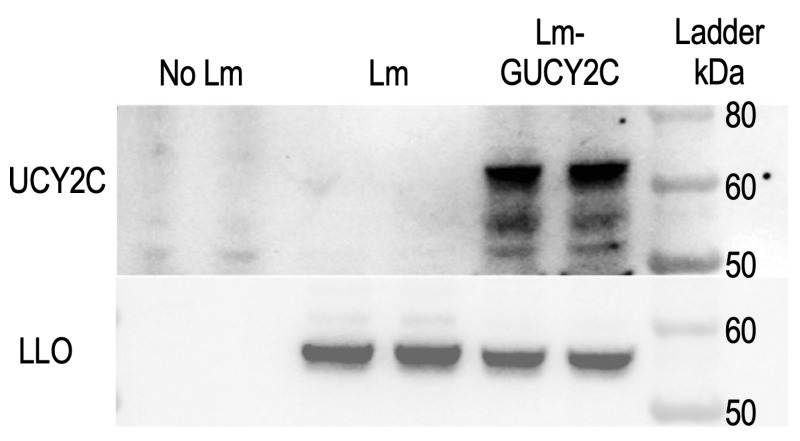
Lm-GUCY2C Functional Testing. J774A.1 cells were infected in duplicate with vehicle, parental Lm, or Lm-GUCY2C. Cell lysates were then examined by immunoblot for GUCY2C and LLO.

To confirm functional antigen expression, J774A.1 murine macrophages, the standard and most widely used cell line for evaluating Lm intracellular infection and antigen expression [20], were infected with Lm-GUCY2C or empty-vector control Lm at an MOI of 10:1. Qualitative Western blot analysis of cell lysates at 6 h post-infection detected a band of the expected molecular weight corresponding to the ActAN100*-Syn18-hGUCY2C_23–429_ fusion protein in Lm-GUCY2C-infected cells (Figure 2 and Figure S3). LLO staining confirmed productive intracellular infection in both Lm-GUCY2C and control conditions. No GUCY2C signal was detected in uninfected cells or parental Lm-infected cells, confirming that GUCY2C expression is cassette-dependent. Additional lower-molecular-weight bands in the GUCY2C blot observed in Lm-GUCY2C-infected lanes likely represent proteolytic processing or degradation products of the fusion protein secreted from the Lm to the J774A.1 cytosol, consistent with the known susceptibility of ActA-fusion proteins to host cell proteases during intracellular infection. Future studies will characterize Lm-GUCY2C infection in human macrophages and longitudinal samples in patients receiving Lm-GUCY2C.

### 3.3. Lm-GUCY2C Elicits Robust Antigen-Specific T Cell Responses In Vivo

**Figure 3:**
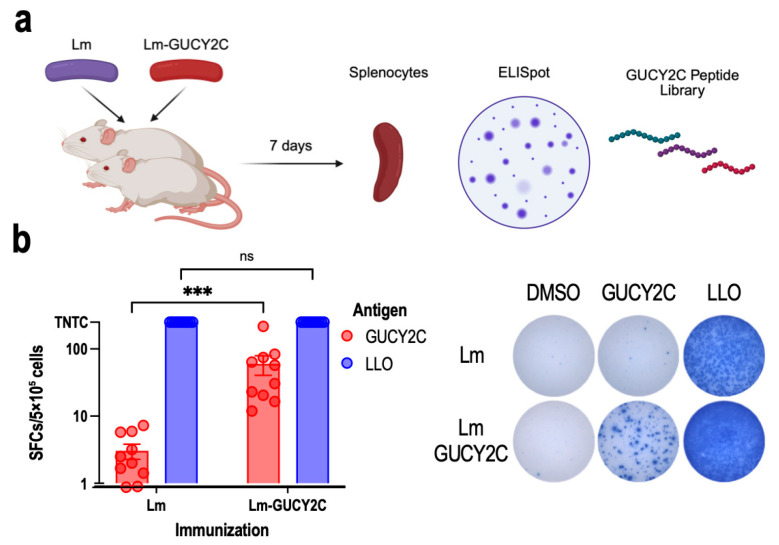
Lm-GUCY2C Immunogenicity in Mice. (**a**) BALB/c mice (n = 10 per group; 5 male, 5 female) received a single i.p. injection of 1 × 10^7^ CFU of Lm-GUCY2C or control Lm. Spleens were harvested at day 7 for IFNγ ELISpot analysis using an overlapping peptide library of human GUCY2C (15-mer in length; 11-mer overlap). (**b**) IFNγ ELISpot results from splenocytes of Lm-GUCY2C- and control Lm-immunized mice stimulated with human GUCY2C peptide library or LLO (positive control). Each symbol represents the antigen-specific response of one mouse; bars indicate mean ± SEM; ns indicates *p* > 0.05, *** *p* < 0.001, Two-way ANOVA. TNTC indicates “too numerous to count”. Representative images of a single well from one animal are shown.

To assess the Lm-GUCY2C in vivo immunogenicity, BALB/c mice (n = 10 per group; 5 male, 5 female) were randomized to receive a single i.p. dose of 1 × 10^7^ CFU of Lm-GUCY2C or control Lm (Figure 3a). Safety was not examined, reflecting differences in human and mouse GUCY2C sequences [14] and prior work demonstrating the safety of Lm expressing mouse GUCY2C in syngeneic systems [21]. IFNγ ELISpot quantifies individual T cells secreting IFNγ in direct response to specific peptide stimulation and is a standard measure of antigen-specific cellular immunity. Here, IFNγ ELISpot analysis of splenocytes stimulated with an overlapping hGUCY2C peptide library revealed robust antigen-specific responses in Lm-GUCY2C-immunized mice, significantly above DMSO background and control Lm-immunized mice, which produced no detectable hGUCY2C-specific response (Figure 3b). There was a trend toward greater responses in females (Supplementary Figure S4), but the study was not powered to detect sex-specific differences. LLO peptide stimulation confirmed splenocyte reactivity and viability in both groups. These data demonstrate that Lm-GUCY2C induces a potent human GUCY2C-specific cellular immune response.

### 3.4. cGMP Manufacturing and Quality Release Testing

Based on preclinical confirmation of genetic integrity and immunogenicity, the lead Lm-GUCY2C clone was transferred to Waisman Biomanufacturing for cGMP production. Pilot-scale process development established the fermentation and downstream processing parameters used for GMP manufacturing of Lm-GUCY2C. Across two pilot runs, Lm-GUCY2C demonstrated consistent exponential growth kinetics in fed-batch fermentation, with harvest-qualifying OD_600_ values of ≥2.0 reached within 3.1–4.5 h of effective fermentation time, consistent with historical Lm fermentation profiles at this facility (Supplementary Figure S5). Seed culture OD at 12 and 16 h was equivalent, and a 12 h seed incubation was adopted. These process parameters were transferred to cGMP production, and a single GMP lot of Lm-GUCY2C drug product was produced and formulated in PBS/10% glycerol at 1.2 × 10^9^ CFU/mL, filled into 2 mL glass cryovials, and stored at −80 °C. Release testing confirmed drug product identity by PCR, potency by CFU enumeration, purity by absence of microbial contamination, etc. (details in Supplementary Table S3). These data confirm that Lm-GUCY2C was successfully manufactured to clinical-grade standards suitable for human administration.

### 3.5. Post-Thaw Stability in Cryovials

**Figure 4:**
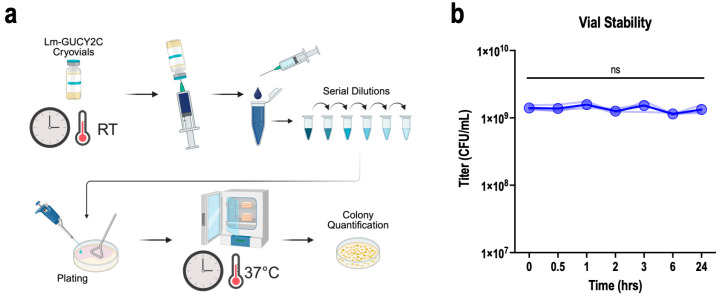
Cryovial Stability. (**a**) Schematic of the cryovial stability workflow. (**b**) Lm-GUCY2C recovery (CFU/mL) from thawed Lm-GUCY2C cryovials kept at room temperature post-thaw. No statistically significant decline was observed across any time interval (One-way repeated measures ANOVA vs. 0 h). Faded lines represent individual replicate cryovials with titer measurements over time; heavy line and symbols indicates means; no comparisons were statistically significant (ns indicates *p* > 0.05).

Post-thaw viability in cryovials was assessed by allowing cGMP Lm-GUCY2C cryovials to thaw at room temperature and then collecting samples over a 24 h time course while the vials remained at room temperature (Figure 4). Samples collected at each time point were plated on TSA plates in serial dilutions to determine Lm-GUCY2C loss. CFU recovery remained consistent across all time points from 0 to 24 h, with no statistically significant decline detected at any interval. These data indicate that the thawed Lm-GUCY2C drug product maintains full viability for at least 24 h under ambient conditions, providing an extensive clinical preparation window.

### 3.6. In-Use Stability in Saline Infusion Bags

**Figure 5:**
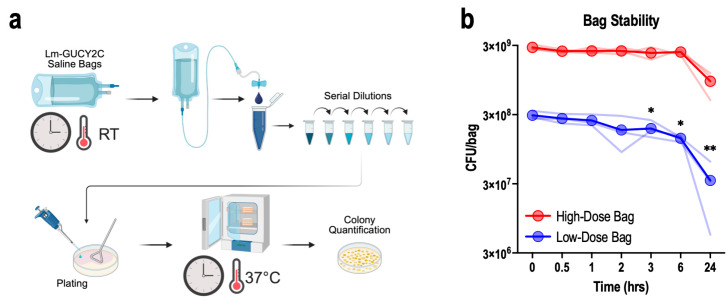
Saline Bag Stability. (**a**) Schematic of saline bag stability workflow showing sample collection via i.v. line. (**b**) CFU recovery from low-dose (3 × 10^8^ CFU/bag) and high-dose (3 × 10^9^ CFU/bag) saline bags over 24 h (computed total CFU/bag based on sample titer). Symbols and heavy lines represent the means, and faded lines indicate the individual values of 3 individual bags titered over time; * *p* < 0.05, ** *p* < 0.01, one-way repeated measures ANOVA vs. 0 h; no comparisons were statistically significant in high-dose bags (**b**).

To ensure stability across a range of clinical dose levels, the stability of Lm-GUCY2C formulated in saline bags for intravenous (i.v.) infusion was assessed for both the highest (3 × 10^9^ CFU/bag) and lowest (3 × 10^8^ CFU/bag) anticipated dose levels in 250 mL saline infusion bags, sampled through a filter-free i.v. line over 24 h (Figure 5a). Bags were prepared by collecting Lm-GUCY2C immediately after thawing cryovials using an appropriately sized syringe and needle and directly transferring it to a 250 mL saline bag via the injection port. Bags were gently hand-mixed for 2 min and then sampled at various time points via a filter-free i.v. line. Prior to each sampling time point, the bag was gently hand-mixed, and the line was flushed with 20 mL of drug product to ensure accurate sampling.

For the high-dose formulation, CFU recovery was maintained consistently from 0 to 6 h post-preparation, with a notable but non-significant decline at 24 h (Figure 5b). For the low-dose formulation, CFU recovery marginally declined at 3 and 6 h, then declined significantly at 24 h (Figure 5b). These data establish a practical in-use window of no more than 2 h for low-dose preparations, after which dose accuracy may be substantially compromised. Given that infusion occurs over 1 h, this suggests that infusion of low-dose preparations should begin within 1 h of preparation.

### 3.7. Closed-System Transfer Device Compatibility Testing

**Figure 6:**
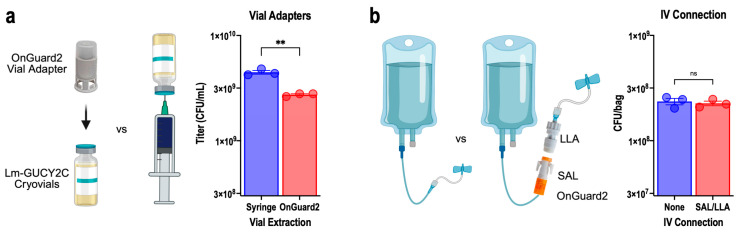
Closed System Compatibility. (**a**) The OnGuard2 vial adapter was compared to a standard needle and syringe for collecting Lm-GUCY2C from cryovials. (**b**) The OnGuard2 SAL and LLA connectors used to connect i.v. lines for administration were compared to standard Luer lock connectors to determine CFU recovery from low-dose (3 × 10^8^ CFU/bag) saline bags prepared as in Figure 5. Symbols represent individual cryovials (**a**) or individual bags (**b**); mean ± SEM; ns indicates *p* > 0.05, ** *p* < 0.01, *t*-test.

Cryovial (Figure 4) and bag stability (Figure 5) studies used a conventional syringe and needle for vial extraction or tubing for bag sampling, respectively, rather than closed systems typical for clinical dispensing and administration of oncology therapies. Here, the OnGuard2 Vial Adapter, Syringe Adaptor Lock (SAL), used to inject vaccine into the infusion bag, and the Luer Lock Adaptor (LLA), used to connect the i.v. line, were evaluated as components of a closed-system i.v. administration setup designed to minimize exposure of healthcare personnel to drug products during preparation and delivery (Figure 6). The OnGuard2 Vial Adaptor, which routes vaccine withdrawal past an activated charcoal filter and a 0.22 μm membrane filter, substantially reduced CFU recovery relative to standard needle-and-syringe transfer (Figure 6a). This reduction is attributable to physical retention of bacteria on the filter membrane. Accordingly, the OnGuard2 Vial Adaptor is contraindicated for use with Lm-GUCY2C. In contrast, low-dose bags prepared without the Vial Adapter and sampled with SAL and LLA adapters (for closed connection of i.v. bags to i.v. lines at bedside infusion) showed CFU recovery comparable to standard open-transfer controls, with no statistically significant difference (Figure 6b). These data confirm that OnGuard2 SAL and LLA connectors are fully compatible with Lm-GUCY2C and can be incorporated into the clinical administration protocol without affecting dose delivery. The outcomes for all evaluated device components are summarized in Table 1.

## 4. Discussion

This study provides a comprehensive translational characterization of Lm-GUCY2C, a GMP-manufactured, attenuated Lm cancer vaccine designed for heterologous boosting of Ad5.F35-GUCY2C-PADRE-primed immunity in colorectal cancer patients. We present construct verification by PCR and Sanger sequencing; in vitro confirmation of antigen expression; in vivo immunogenicity in mice; GMP manufacturing and release testing; and the first systematic characterization of in-use stability and i.v. device compatibility for an Lm-based cancer vaccine product.

The stability findings carry direct, actionable consequences for clinical protocol design. The cryovial stability data confirm that post-thaw preparation is not a rate-limiting constraint: viability was fully maintained for 24 h under ambient conditions. This window easily accommodates the typical clinical pharmacy preparation and dose-check workflow without requiring dedicated refrigerated staging. Similarly, high-dose i.v. bag preparations (3 × 10^9^ CFU/bag) maintained CFU recovery for 3 h in saline, providing flexibility in scheduling administration. In contrast, low-dose preparations (3 × 10^8^ CFU/bag) exhibited some losses beginning at 2 h, establishing a 2 h in-use window. This dose-dependent difference in stability most likely reflects concentration-dependent surface adsorption to infusion bag materials (polyvinyl chloride; PVC). PVC-based i.v. bags and tubing are known to cause clinically significant adsorptive losses of biologics [31], and bacterial adhesion to PVC medical device surfaces is well established [25,26]. At lower bacterial density, a greater proportion of cells may contact and adhere to hydrophobic PVC surfaces, amplifying proportional dose loss. Indeed, concentration-dependent stability effects have been noted for other live biologic products [32].

The device compatibility data are equally actionable for clinical implementation. The OnGuard2 SAL and LLA connectors provide meaningful occupational safety benefits for pharmacy and nursing staff handling a live bacterial product and are fully compatible with Lm-GUCY2C without CFU loss, a result that supports their routine use in the clinical administration setup. However, the OnGuard2 Vial Adaptor is contraindicated. Its integrated 0.22 μm membrane filter and activated charcoal filter physically retain bacteria, causing substantial dose loss before the product reaches the infusion bag. This result underscores a general principle for live-organism drug products: any device component incorporating membrane filtration or adsorptive materials must be prospectively validated before use with live biological therapies. Similarly, inline i.v. filters of any type must be avoided for delivery of live bacterial products, as bacterial retention on such filters would deliver a substantially reduced dose to the patient.

Our translational findings complement a sparse literature on stability of live bacterial vaccine products in clinical administration formats. While attenuated bacterial vaccines for infectious disease (e.g., BCG, oral typhoid Ty21a) have established some handling precedents, no equivalent data have been published for any Lm-based cancer vaccine. The current work establishes a model characterization framework with systematic evaluation of vial stability, bag stability across clinically relevant dose levels, and component-by-component device assessment, which should be adopted for future live bacterial immunotherapy products entering clinical development. The complete genetic and functional characterization described here also establishes the translational readiness of Lm-GUCY2C as a clinical candidate. Confirmation of integration site fidelity and retention of the ΔactAΔinlB safety deletions, combined with in vitro expression data and in vivo immunogenicity, meets contemporary regulatory expectations for preclinical evaluation to support clinical testing. However, some limitations should be noted. The stability data derive from a single GMP lot. All experiments were performed at room temperature (~22 °C); temperature excursion protocols should be defined for clinical use and validated separately. Although compatibility with the specific OnGuard2 SAL and LLA components was established, other closed-system transfer device brands and configurations require independent validation prior to use.

## 5. Conclusions

We describe the end-to-end translational characterization of Lm-GUCY2C, a GMP-manufactured attenuated Lm cancer vaccine expressing human GUCY2C for clinical use in colorectal cancer patients. Construct integrity and in vitro and in vivo functionality were confirmed. Post-thaw stability and in-use device compatibility characterization established that: (i) drug product is highly stable in cryovials post-thaw; (ii) drug product diluted in saline i.v. bags must be administered within 2 h; (iii) OnGuard2 SAL and LLA connectors are compatible; and (iv) OnGuard2 Vial Adaptor and inline filtered i.v. lines are contraindicated. Together, these data provide the preclinical and translational foundation, as well as the clinically actionable handling guidelines required to advance Lm-GUCY2C to human clinical testing.

## Figures and Tables

**Table 1:** Summary of device component compatibility with Lm-GUCY2C.

Device Component	Application	Outcome	Compatible?
OnGuard2 Vial Adaptor	Vaccine withdrawal from vials	Significant CFU loss	No
Sterile syringe & needle	Vial-to-bag transfer	No CFU loss	Yes
IV line with inline filter	Bag-to-patient delivery	Significant CFU loss expected	No
Filter-free IV infusion line	Bag-to-patient delivery	No CFU loss	Yes
OnGuard2 Syringe Adaptor Lock (SAL)	Closed-system syringe adaptor lock for IV-line connection	No CFU loss	Yes
OnGuard2 Luer Lock Adaptor (LLA)	Closed-system Luer Lock port connector	No CFU loss	Yes

## Data Availability

The raw data supporting the conclusions of this article will be made available by the authors on request.

## Supplementary Materials

The following supporting information can be downloaded at: https://www.mdpi.com/article/10.3390/vaccines14050461/s1, Table S1: Identity PCR Primers; Table S2: In-Use Stability and Device Compatibility Testing Materials; Figure S1: Full Gel Images and Quantification for Figure 1; Figure S2: Lm- GUCY2C Whole-Genome Sequencing Confirmation; Figure S3: Full Blot Images and Quantification for Figure 2; Figure S4: Sex Differences in Lm- GUCY2C-Immunized Mice; Figure S5: Pilot Lm-GUCY2C Fermentation Data; Table S2: Selected Release Testing Results.
