# Supplementary material for: In-Use Stability and Device Compatibility Define Clinically Actionable Handling Limits for a GMP-Produced Attenuated Listeria monocytogenes Vaccine Expressing GUCY2C
Source: Vaccines (Basel). 2026 May 21;14(5):461. doi: 10.3390/vaccines14050461 (PMC13211327; doi:10.3390/vaccines14050461)
Supplement: Supplementary file 1 [file vaccines-14-00461-s001.zip › vaccines-4308156-supplementary.pdf]

Supplementary Data

Supplementary Table S1. Identity PCR Primers.

Primer Pair	Sequence (5′-3′)	Target	Lm- GUCY2C Expected Size	Wildtype Lm Expected Size
1	GACGTCATTAACCCTCAC<br>CAACAGTACTGCGATGAGTG	Cassette promoter to *CAT*	2467 bp	No band
2	GTGATACGCCTATATAGGTTAATGTC<br>CGGTTCAGGGCAGGGTCG	Cassette promoter to GUCY2C	2372 bp	No band
3	GAGAAAGGTGGATCTGCAAG<br>CAACAGTACTGCGATGAGTG	Syn18x5 to *CAT*	1956 bp	No band
4	CGGTTCTTACGAAATCTCTG<br>CAACAGTACTGCGATGAGTG	GUCY2C to *CAT*	1426 bp	No band
5	GCTTCTGTTTCTTACTTCTCTC<br>TGACCGAATAATAAGATACCG	GUCY2C	371 bp	No Band
6	AGCAATGGGAACTCCTGGTG<br>ATGCAGTGACAAATGTGCCG	*hly*	917 bp	917 bp
inlB	AGTAGGAACTGCAATGGCTCT<br>GAACTGATCCGTTTTCAGCGA	*inlB*	464 bp	2091 bp
actA	TAGCGTATCACGAGGAGGGA<br>GAGCTGCGGGTGTTTGTAAG	*actA*	216 bp	2086 bp

Supplementary Table S2. In-Use Stability and Device Compatibility Testing Materials.

Materials	Vendor	Catalog No.
1 mL syringes	BD Biosciences	309628
3 mL Luer lock syringe	BD Biosciences	309657
23g x 1 BD precision glide needle	BD Biosciences	305145
Saline bags	Baxter	2B1322
IV lines without filters	Baxter	2R8875
OnGuard2 Syringe Adaptor Lock (SAL)	B Braun	412174
OnGuard2 Luer Lock Adaptor (LLA)	B Braun	412160
OnGuard2 Vial Adaptor	B Braun	412161

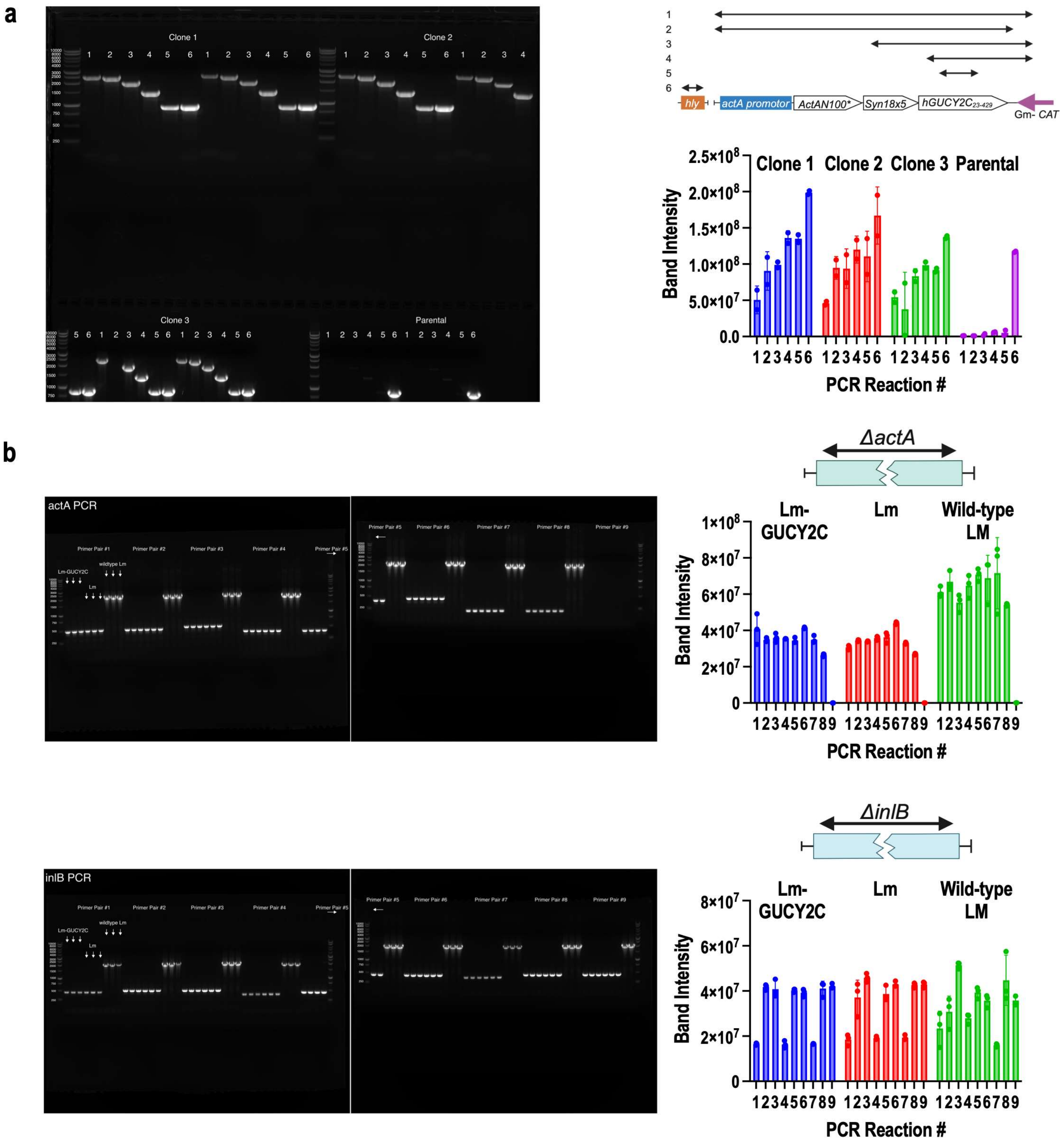

Supplementary Figure S1. Full Gel Images and Quantification for Figure 1. (**a**) Full gel image and quantification of Lm and Lm-GUCY2C examined with various PCR primer pairs specific to the ActA-hGUCY2C expression cassette (#1-#5) and a pair specific to the Lm gene *hly* (#6); the image corresponds to **Figure 1c** of the manuscript. For each clone, replicate PCRs were run (grouped by replicate). Band intensity quantification of each replicate PCR for each clone is shown. (**b**) Full gel image and quantification of truncated actA (top) and inlB (bottom) genes in Lm-GUCY2C and in parental ΔactA/ΔinlB Lm, and the larger wild-type genes in wild-type Lm. Nine different primer pairs were tested for each gene (actA pair #9 failed to amplify). For each primer pair, triplicate reactions were run on each culture (grouped by primer pair). The image corresponds to **Figure 1d** of the manuscript.

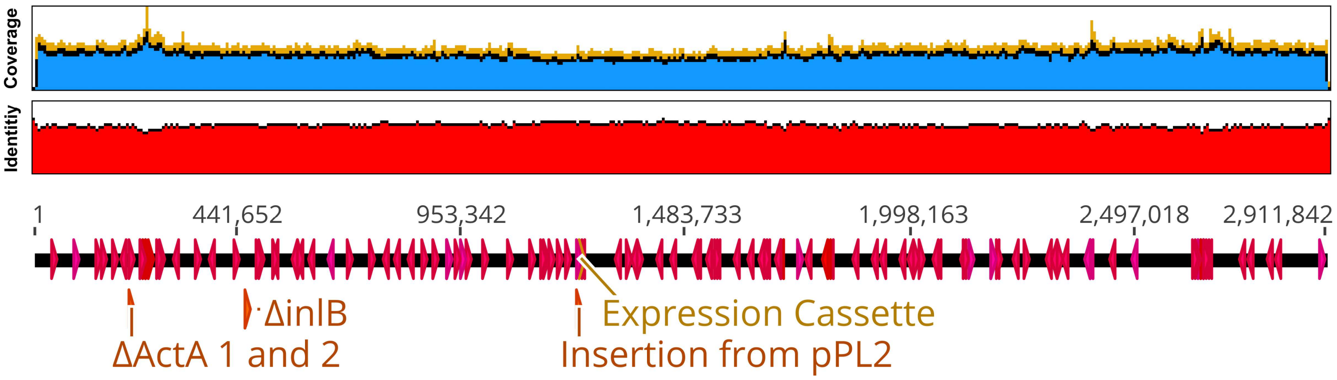

Supplementary Figure S2. Lm-GUCY2C Whole Genome Sequencing Confirmation. The lead Lm-GUCY2C clone identified for GMP manufacturing underwent whole genome sequencing, and reads were aligned to the predicted sequence, revealing high coverage and identity to the expected sequence. Numbers indicate bp of the nearly 3M bp Lm-GUCY2C genome. Most features are too abundant to indicate, but tRNA sites, ΔactA and ΔinlB sites, and the pPL2 insertion, including ActA-hGUCY2C expression cassette, are indicated.

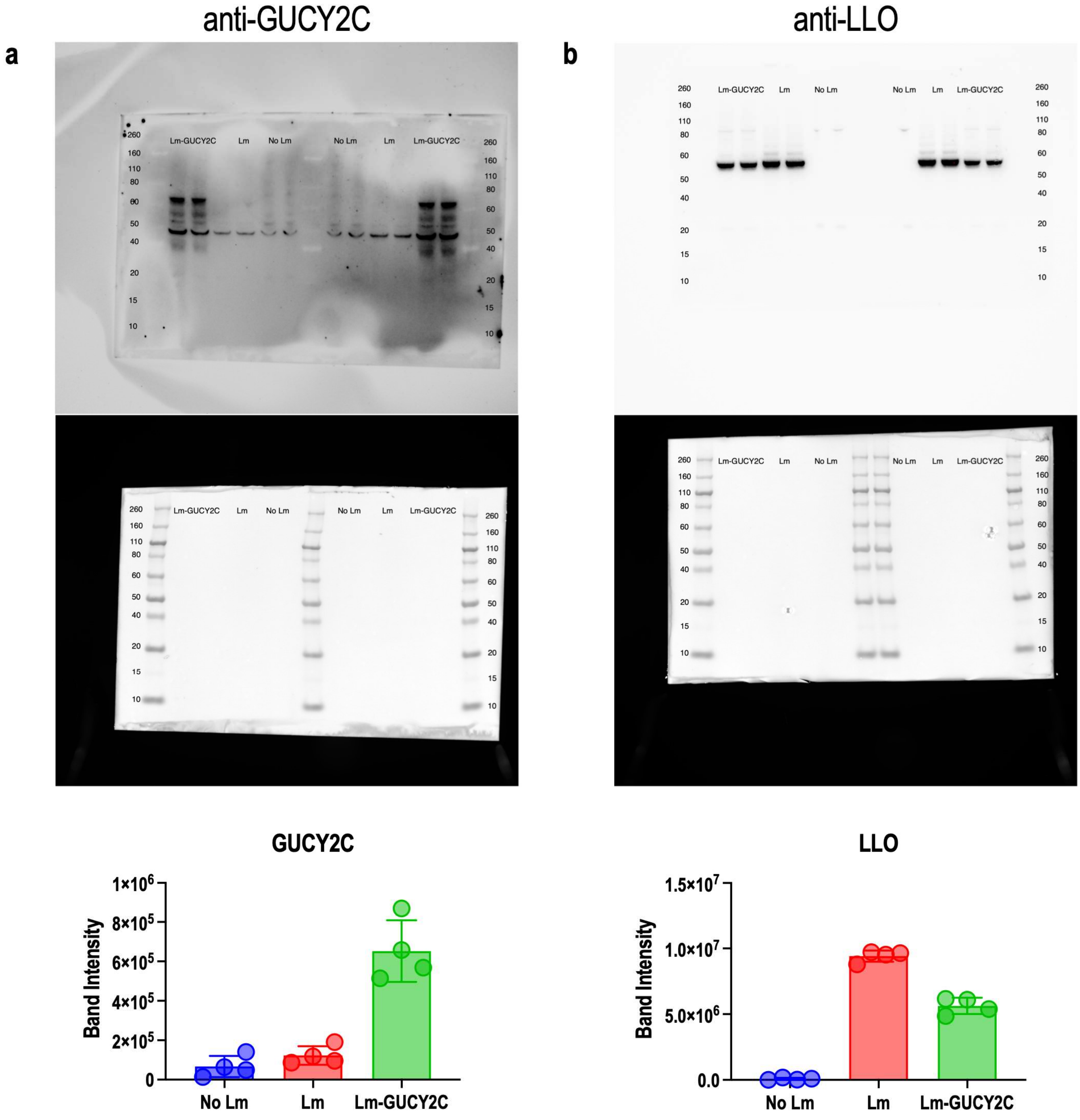

Supplementary Figure S3. Full Blot Images and Quantification for Figure 2. J774A.1 cells were infected with vehicle (no Lm), parental Lm, or Lm-GUCY2C. Cell lysates were then examined by immunoblot for GUCY2C (**a**) and LLO (**b**). The top images indicate bioluminescence images, and the bottom images indicate white light images for GUCY2C (**a**) and LLO (**b**) immunoblots. The images and data correspond to **Figure 2** of the manuscript.

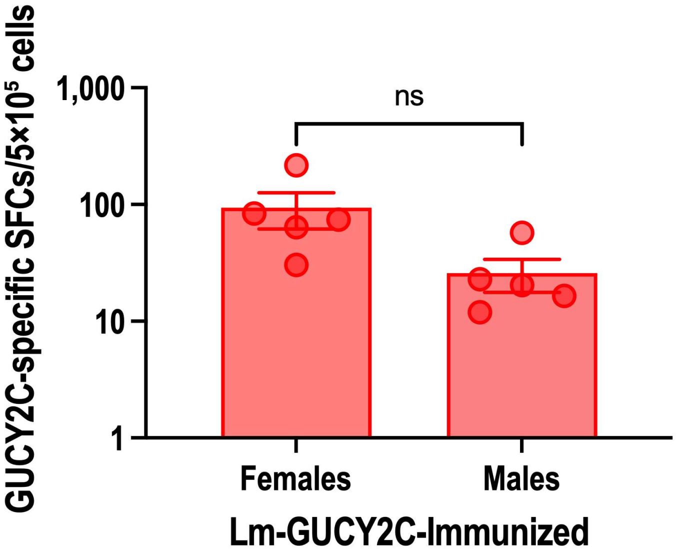

Supplementary Figure S4. Sex Differences in Lm-GUCY2C-Immunized Mice. Data indicate GUCY2C-specific responses in **Figure 3b**, stratified by sex. Each symbol represents the antigen-specific response of one mouse; bars indicate mean ± SEM; t-test.

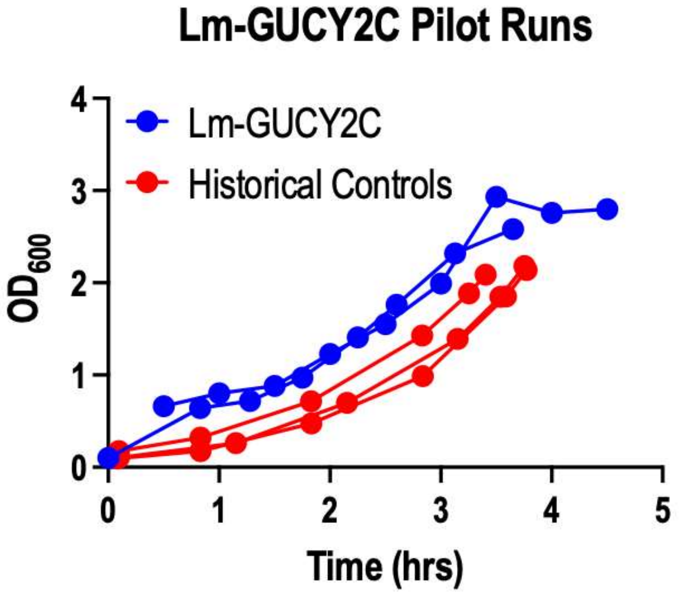

Supplementary Figure S5. Pilot Lm-GUCY2C Fermentation Data. Lm-GUCY2C growth in two pilot runs are shown in comparison to 3 historical runs of different Lm products.

Supplementary Table S3. Selected Release Testing Results.

Test	Specification	Test Result	Pass/Fail
pH	7.0 ± 1.0	6.7	PASS
Osmolality	Report Result	1766 mOsm/kg	PASS
Visual Inspection	Opaque, white to off-white suspension with no visible foreign particulates	Opaque, white to off-white suspension with no visible foreign particulates	PASS
Endotoxin	< 0.5 EU/mL	< 0.5 EU/mL	PASS
Western Blot for GUCY2C	Principle band ~70 kDa	Principle band ~70 kDa	PASS
Lm Identity by PCR – *actA*/*inlB* deletions	Confirmed deletion by PCR - *actA* band ~216 bp, *inlB* band ~464 bp	Confirmed deletion by *actA* band ~216 bp and *inlB* band at ~464 bp	PASS
Lm Identity by PCR – GUCY2C Expression Cassette	Confirm presence by PCR - dominant bands at ~2372 bp, ~1956 bp, and ~906 bp	Confirm presence by PCR - dominant bands at ~2372 bp, ~1956 bp, and ~906 bp	PASS
Microbial Examination for Specified Microorganisms	Absence for<br>*S. aureus*<br>*P. aeruginosa*<br>*E. coli*<br>*Salmonella*<br>*C. albicans*<br>*B. fragilis*<br>*Clostridia*<br>*A. brasiliensis*	Absence for<br>*S. aureus*<br>*P. aeruginosa*<br>*E. coli*<br>*Salmonella*<br>*C. albicans*<br>*B. fragilis*<br>*Clostridia*<br>*A. brasiliensis*	PASS
Identity by MALDI-TOF-Mass Spec	Confirm host species *L. monocytogenes*	Confirm host species *L. monocytogenes*	PASS
